# Assessing Revisit Risk in Emergency Department Patients: Machine Learning Approach

**DOI:** 10.2196/74053

**Published:** 2025-08-07

**Authors:** Wang-Chuan Juang, Zheng-Xun Cai, Chia-Mei Chen, Zhi-Hong You

**Affiliations:** 1 Quality Management Center, Kaohsiung Veterans General Hospital, No.386, Dazhong 1st Rd., Zuoying Dist., Kaohsiung, 813414, Taiwan, 886 7-342-2121 ext 4191; 2 Department of Business Management, College of Management, National Sun Yat-sen University, Kaohsiung, Taiwan; 3 Department of Health-Business Administration, Fooyin University, Kaohsiung, Taiwan; 4 Department of Information Management, College of Management, National Sun Yat-sen University, Kaohsiung, Taiwan

**Keywords:** unscheduled return visit, machine learning, electronic health records, deep learning, clinical decision support system

## Abstract

**Background:**

Overcrowded emergency rooms might degrade the quality of care and overload the clinic staff. Assessing unscheduled return visits (URVs) to the emergency department (ED) is a quality assurance procedure to identify ED-discharged patients with a high likelihood of bounce-back, to ensure patient safety, and ultimately to reduce medical costs by decreasing the frequency of URVs. The field of machine learning (ML) has evolved considerably in the past decades, and many ML applications have been deployed in various contexts.

**Objective:**

This study aims to develop an ML-assisted framework that identifies high-risk patients who may revisit the ED within 72 hours after the initial visit. Furthermore, this study evaluates different ML models, feature sets, and feature encoding methods in order to build an effective prediction model.

**Methods:**

This study proposes an ML-assisted system that extracts the features from both structured and unstructured medical data to predict patients who are likely to revisit the ED, where the structured data includes patients’ electronic health records, and the unstructured data is their medical notes (subjective, objective, assessment, and plan). A 5-year dataset consisting of 184,687 ED visits, along with 324,111 historical electronic health records and the associated medical notes, was obtained from Kaohsiung Veterans General Hospital, a tertiary medical center in Taiwan, to evaluate the proposed system.

**Results:**

The evaluation results indicate that incorporating convolutional neural network–based feature extraction from unstructured ED physician narrative notes, combined with structured vital signs and demographic data, significantly enhances predictive performance. The proposed approach achieves an area under the receiver operating characteristic curve of 0.705 and a recall of 0.718, demonstrating its effectiveness in predicting URVs. These findings highlight the potential of integrating structured and unstructured clinical data to improve predictive accuracy in this context.

**Conclusions:**

The study demonstrates that an ML-assisted framework may be applied as a decision support tool to assist ED clinicians in identifying revisiting patients, although the model’s performance may not be sufficient for clinic implementation. Given the improvement in the area under the receiver operating characteristic curve, the proposed framework should be further explored as a workable decision support tool to pinpoint ED patients with a high risk of revisit and provide them with appropriate and timely care.

## Introduction

### Background

Health care services are inherently risky, as they might involve unpredictable events. Risks may damage a health care provider’s finances, patient safety, staff satisfaction, or liabilities. Risk management in health care is extremely important—as human lives are on the line—which consists of a complex set of clinical and administrative systems, processes, procedures, and reporting structures designed to monitor, identify, assess, mitigate, or prevent risks to patients.

The coordination between primary care providers and hospitals in Taiwan often lacks a streamlined and effective referral mechanism, contributing significantly to emergency department (ED) overcrowding. In Taiwan, patients often seek medical support from major medical institutions rather than local clinics. This tendency may be attributed to the structure of the national health care insurance system in Taiwan, where diagnostic fees are similar between local clinics and large medical institutions. In the current system, patients with nonemergency conditions frequently bypass local clinics and primary care providers, opting to visit the ED directly. Moreover, primary care providers may fail to appropriately direct patients to specialized departments or hospitals due to limited communication channels, inadequate follow-up procedures, or the absence of clear referral protocols. Without a robust referral system, conditions that could be managed effectively in primary care settings are unnecessarily escalated to emergency care, further straining ED resources.

Premature self-discharge, where patients choose to leave the ED before completing their prescribed treatment or before being officially discharged by medical professionals, is a significant contributing factor to ED overcrowding in Taiwan. This behavior often stems from patients’ subjective perception of their condition improving or from a lack of understanding of the importance of completing treatment. Cultural attitudes toward health and limited medical knowledge further exacerbate this issue, as patients may misinterpret temporary symptom relief as a full recovery. Such self-discharges frequently result in incomplete or interrupted care, which can lead to complications or the progression of the underlying medical condition. Consequently, these patients are likely to return to the ED when their symptoms reappear or deteriorate, thereby increasing the number of unscheduled return visits (URVs). This not only strains ED resources but also disrupts patient flow and increases the workload for health care providers. Additionally, premature self-discharge may be influenced by nonmedical factors, such as long waiting times, perceived inconvenience, or economic pressures, despite Taiwan’s universal health care system. Patients who prioritize immediate symptom relief over long-term health outcomes may also undervalue the importance of follow-up care and professional medical advice.

Patients admitted to the ED often require timely health care resources. A growing number of ED patients in recent years demand more health care resources than ever [1]; overcrowded emergency rooms are a common phenomenon worldwide. According to Taiwan’s ED statistical data [2], more than 6.1 million ED visits in 2022 contributed to a 13.6% increase compared with 2021. Among the ED admissions, patients with varying severity of diseases or comorbidities request even more health care services. Efficient use of ED services becomes a challenge for health care management, where the unexpected ED revisit rate is a common performance metric. The number of revisits could be reduced if a clinic support system assesses the revisit risk at a patient’s initial visit to the ED.

Addressing the issue of ED overcrowding requires the implementation of long-term solutions aimed at optimizing health care resource allocation and improving patient flow management. While systemic changes, such as enhancing primary care accessibility and strengthening referral mechanisms, are critical, such reforms often require significant time and policy adjustments. To provide immediate and practical relief, this study focuses on the development of a clinical decision support system designed to predict the likelihood of patient unplanned revisits. By leveraging predictive analytics, the proposed clinical decision support system enables health care providers to identify high-risk patients and implement targeted interventions, such as personalized discharge planning and follow-up care. These measures not only improve patient outcomes but also help alleviate the burden on EDs, offering a scalable and effective tool to mitigate overcrowding while broader systemic solutions are pursued.

The applications of information technology to improve health care delivery have been appreciated for decades. To control risks and reduce clinical errors, health care organizations can learn from retrospective events. Health information technologies, such as electronic health records (EHRs), provide good access to retrospective patient information. With the expanding applications of machine learning (ML) on outcome prediction, these use cases have demonstrated the applicability of assessing patient risk from data repositories. Therefore, this study aims to develop an ML-assisted model that predicts URVs in the ED and anticipates that such an application could improve patient safety and reduce medical costs by decreasing the frequency of URVs.

### Related Work

It is critical for emergency clinicians to determine high-risk patients who might return in worse conditions or even die. A study [3] on predictors of 30-day ED revisits and 90-day functional decline or mortality concluded that age, sex, polypharmacy, and cognitive impairment were independent predictors of a 30-day ED revisit and that no effective clinical prediction model could be developed. Another study [4] analyzed multi-state ED revisit data. Within 3 days of an index ED visit, 8.2% of patients returned within 72 hours; 32% of those revisits occurred at a different health care institution. Revisit rates varied by diagnosis and by state. Research on ED revisits in different countries yielded varying findings. To differentiate patient risk groups, an analytic study [5] collected 10-year EHR data from multiple health care institutes and applied group-based trajectory modeling. Patients with behavioral diagnoses, injuries, alcohol and substance abuse, stroke, or diabetes had a higher risk of revisiting.

Several studies [6-8] analyzed ED revisit cases in Taiwan. The first study concluded the following findings: 5.47% of patients had a revisit within 72 hours; most revisits were related to illness; and abdominal pain was the most common presentation (55.7%). The second analysis, focusing on unplanned revisits with abdominal pain, demonstrated that older patients receiving multiple analgesics and laboratory tests had a high risk of URV. The third work summarized factors that may impact ED revisits, including blood pressure, pulse rate, fever, triage level, gender, and main illness, while old age was identified as a key factor. The fourth work investigated the risk factors of ED revisits among patients younger than 50 years and applied the decision tree (DT) to identify the variables capable of partitioning the groups into URVs and non-URVs. They found that the Charlson Comorbidity Index (CCI) scores for URVs are higher than those of non-URVs, and the triage levels of URVs are more severe than those of non-URVs.

A prior study [9] analyzed 48-hour ED revisits in a hospital in Thailand. In addition to their revisits mostly being related to gastrointestinal illness (28.76%), they observed the key predictive factors similar to Taiwan’s previous research. The past work encouraged further study to evaluate the most common and critical causes of revisits to improve revisit prediction. Most of the past research analyzed URV cases, excluding non-URV cases, and then applied statistical approaches to determine the common factors of URV patients. Even though most ED patients were non-URVs, they might exhibit some similar clinical characteristics to URV cases. Modern ML models might be able to learn the correlations among those features.

A review study [10] explored the existing research that applied ML models to predict ED revisits. Logistic regression (LR) is the most widely used method, while extreme gradient boosting generally exhibits superior performance. Developing ML prediction models for ED URVs is feasible; however, improving the accuracy beyond 0.75 remains a challenge.

Vest and Ben-Assuli [11] applied a DT algorithm to predict the risk of 30-day ED readmissions. Social determinants of health measurements have poor discriminating ability, but the prediction performance improves with more patient information, including current triage and historical data. McCusker et al [12] designed a screening tool with 27 self-report screening questions to assess health risks in senior patients during the 6 months following their initial ED visits. Davazdahemami et al [13] adopted a deep neural network model to predict URVs to the ED. They applied the word embedding model (Doc2Vec) to encode unstructured physician notes and concluded that leveraging structured and unstructured EHR data improves prediction performance.

## Methods

### Study Population and Setting

Kaohsiung Veterans General Hospital (KVGH) is one of Taiwan’s largest general hospitals serving the southern region of Taiwan and offering inpatient, outpatient, and ED health care services. This study analyzed the retrospective administrative medical data of outpatients who sought ED services at KVGH between January 2018 and December 2022. The studied data contains 2 parts, structured and unstructured data, where structured data refers to EHR data and unstructured data consists of subjective, objective, assessment, and plan notes.

**Figure 1.**
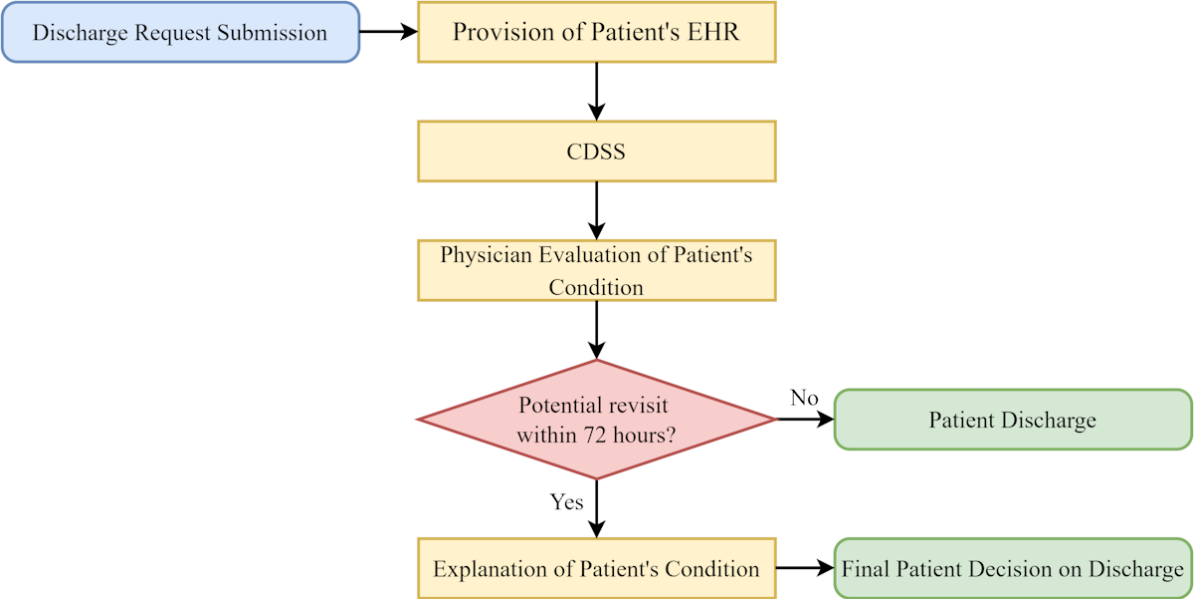
A patient discharge workflow. CDSS: clinical decision support system; EHR: electronic health record.

Figure 1 outlines the workflow when a patient submits a discharge request. In the initial stage, the system retrieves the patient’s medical records and analyzes them in the subsequent stage. The model then assesses the likelihood of the patient revisiting the ED within 72 hours based on their medical data. The prediction outcomes serve as decision support for physicians in the clinical assessment process. If no significant risk factors for readmission are identified, the discharge request is approved. However, if the model predicts a high probability of an unplanned revisit, physicians may advise the patient to stay and provide an explanation of their medical condition. Tables 1-3 outline the demographics of the studied dataset.

**Table 1.** The demographic of the numeric variables in the studied dataset.

Variables	Mean (SD)	Median (range)
Age (years)	53.90 (21.02)	55 (17-111)
HBP^a^	134.14 (23.96)	133 (12-279)
LBP^b^	80.55 (14.88)	80 (6-227)
MAP^c^	98.50 (16.34)	97.67 (17-230)
Pulse	79.91 (15.57)	78 (5-275)
Temperature	36.60 (0.76)	36.50 (25-44)
Breath	17.53 (2.36)	18 (1-79)
SPO_2_	97.43 (3.30)	98 (51-100)
Hospitalization hours	5.07 (5.56)	2.75 (0‐24)
Number of lab results	17.25 (10.79)	20 (0‐75)
Number of medicine	4.04 (2.66)	4 (0‐26)

a HBP: high blood pressure.

b LBP: low blood pressure.

c MAP: mean arterial pressure.

**Table 2.** The demographic of the temporal variables in the studied dataset.

Variables	Range
Admission time	January 1, 2018 (12:01 AM), to December 31, 2022 (11:43 PM)
Discharge time	January 1, 2018 (1:07 AM), to January 2, 2023 (3:59 PM)
Measure time	January 1, 2018 (1:01 AM), to March 16, 2023 (3:17 PM)
Medication begin time	January 1, 2018 (12:25 AM), to January 2, 2023 (11:47 AM)
Medication end time	January 1, 2018 (12:26 AM), to January 23, 2023 (11:59 PM)
Order check time	January 1, 2018 (12:00 AM), to January 4, 2023 (11:43 AM)

**Table 3.** The demographic of the categorical variables in the studied dataset.

Variables	Categories
TTAS^a^	Resuscitation, emergent, urgent, less urgent, and nonurgent
Visit disposition	ED^b^, admission, outpatient, transfer out, and other
Medication frequency	statstat, urgent, once, stat, regular, oncedown, statstdn, and statdown
*ICD-10* ^c^	A00-B99, C00-D49, D50-D89, E00-E89, F00-F99, G00-G99, H00-H59, H60-H95, I00-I99, J00-J99, K00-K95, L00-L99, M00-M99, N00-N99, O00-O9A, P00-P96, Q00-Q99, R00-R99, S00-T88, V00-Y99, Z00-Z99, and U00-U85

a TTAS: Taiwan Triage Acuity Scale.

b ED: emergency department.

c *ICD-10*: *International Statistical Classification of Diseases, Tenth Revision*.

### Prediction Target

The collected data were divided into 2 cohort groups: URV patients and non-URV patients. Our prediction target is a binary indicator of 72-hour URV (coded as URV/non-URV) in ED. Each visit is labeled with an associated indicator column to indicate whether the patient had an ED revisit within 72 hours.

### Ethical Considerations

This study was conducted in accordance with the principles outlined in the Declaration of Helsinki and received approval from the Institutional Review Board (IRB) of KVGH with IRB certification number KSVGH23-CT5-04 (date of approval: October 31, 2023). The requirement for consent to participate was waived by the IRB of KVGH, as the research uses historical electronic medical records without any direct patient intervention. All data points have been deidentified, and no specific individual can be identified from the data. The rights and interests of the patients are not harmed, and this study does not have any impact on patients’ treatment and medication before and after analyzing the data.

### Model Development

**Figure 2.**
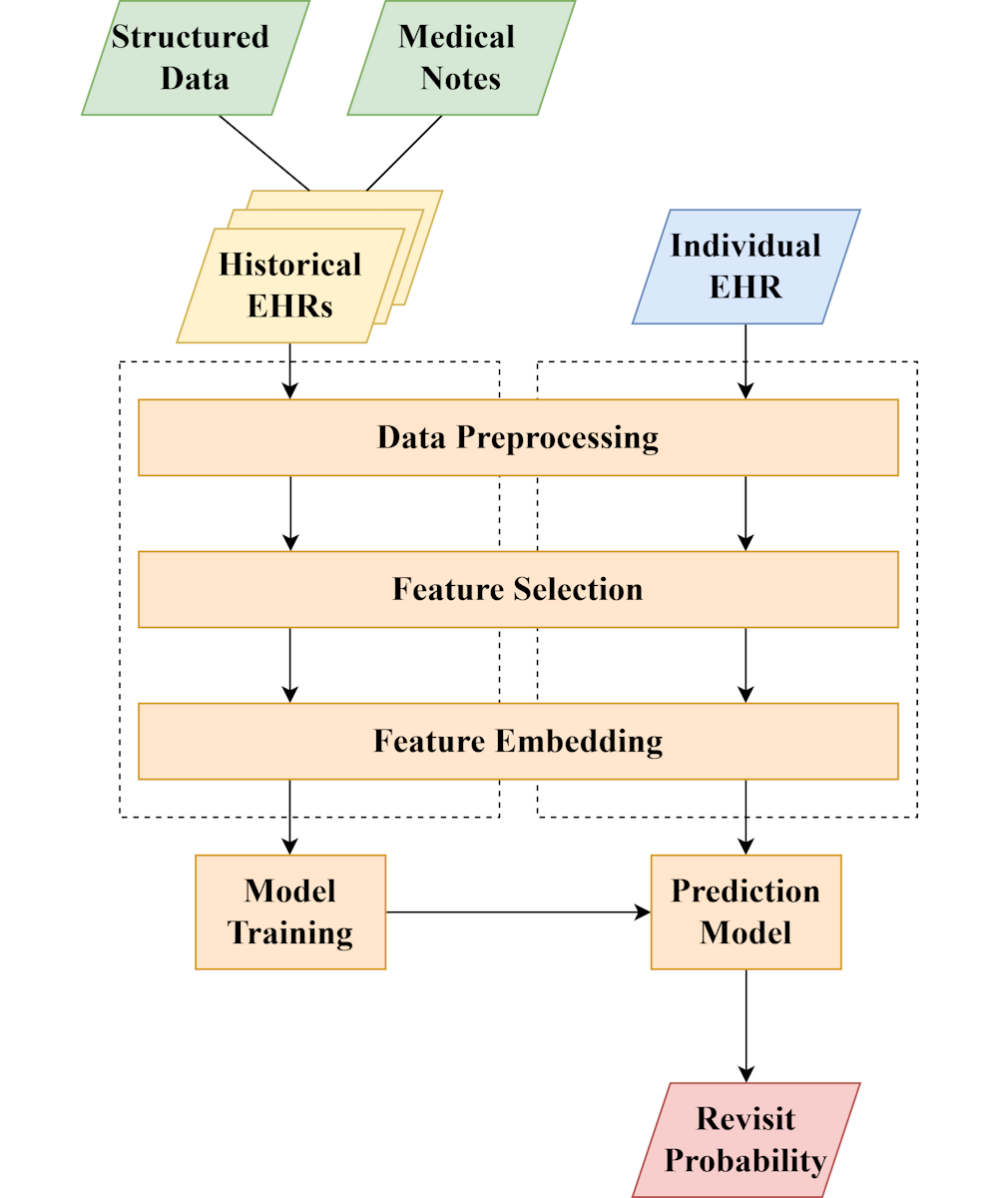
The architecture of the proposed framework. EHR: electronic health record.

Figure 2 outlines the proposed system architecture consisting of the following modules: data preprocessing, feature selection, feature embedding, and prediction model. Each is explained in the following sections.

### Data Preprocessing

The studied data consists of 2 parts: structured and unstructured data. This module performs data cleansing and normalization tasks, removing missing fields and out-of-range data and subsequently normalizing data into a consistent range. Variable values within the same range avoid the problem of excessive weight on certain variable values and then improve the performance of model training. According to preliminary studies, *z* score standardization performs better than Min-Max normalization. This study applies *z* score standardization to normalize and redistribute all pathological feature values to the same interval.

### Model Variable Selection

Model variables, aka features, provide information for the model, and ML algorithms can automatically determine feature weights during the training process. Typically, incorporating more variables can enhance performance. However, irrelevant variables may negatively affect prediction accuracy. Therefore, in real-world applications, feature selection or model variable selection is a critical process for identifying relevant ones to construct an effective prediction model. Without loss of generality, this research adopts the term feature to describe the model variable.

The studied dataset comprises 24 distinct features, including both structured and unstructured data. Structured data are derived from EHR, while unstructured data contents originate from clinicians’ medical notes. To appropriately integrate unstructured data, subjective complaints recorded in medical notes are transformed into corresponding *ICD-10* (*International Statistical Classification of Diseases, Tenth Revision*) codes. This mechanism converts unstructured medical notes into structured data while preserving their original information.

This study defines 3 feature sets (FSs), denoted as FS_A_, FS_B_, and FS_C_. FS_A_ contains all features in the dataset to evaluate model performance without applying any feature selection procedure. Based on preliminary experiments, features related to lab orders and medical orders were found to have no positive impact on the prediction model and were thus excluded from both FS_B_ and FS_C_. To further analyze the effect of incorporating unstructured data on model performance, features derived from unstructured data were removed from FS_B_. Table 4 provides a summary of the features in these 3 sets.

**Table 4.** Feature description.

Feature	Type	FS_A_^a^	FS_B_	FS_C_
Basic ED^b^ visit record
Gender	Cat	✔		✔
Age	Num	✔	✔	✔
Admission time	Num	✔		
Discharge time	Num	✔		
*ICD-10*^c^	Cat	✔		✔
TTAS^d^	Cat	✔		✔
Hospitalization days	Num	✔		
Visit disposition	Cat	✔		✔
Vital Sign				
Patient ID	Cat	✔	✔	✔
Case number	Cat	✔		
Measure time	Num	✔		
HBP^e^	Num	✔	✔	✔
LBP^f^	Num	✔	✔	✔
Pulse	Num	✔	✔	✔
Temperature	Num	✔		✔
Breath	Num	✔		
Lab Order				
Order Check Time	Num	✔		
Lab test name	Cat	✔		
Lab result	Num	✔		
Medication Order				
Medication begin time	Num	✔		
Medication end time	Num	✔		
Medicine	Cat	✔		
Dosage	Num	✔		
Frequency	Cat	✔		

a FS: feature set.

b ED: emergency department.

c ICD-10: International Classification of Diseases-10.

d TTAS: Taiwan Triage Acuity Scale.

e HBP: high blood pressure.

f LBP: low blood pressure.

It is important to note that the patient ID is deidentified and serves to indicate a specific patient, while the case number is a serial identifier for an ED visit. Both features are excluded from the prediction model during training.

ED visits are listed in chronological order. For structured data, each visit is characterized by the following types of features: (1) basic ED visit record, (2) vital signs, and (3) care order information (as outlined in Table 4). The basic ED visit record includes gender, age, *ICD-10*, admission time, discharge time, visit disposition, and triage level coded as a categorical variable based on the type and severity of initial presenting signs and symptoms using the Taiwan Triage and Acuity Scale, ranging from 1 (resuscitation) to 5 (nonurgent). The vital signs include body temperature, respiratory rate, pulse, high blood pressure (HBP), low blood pressure, mean arterial pressure, and oxygen saturation. The care order information includes laboratory tests and medication-related information.

Past studies have revealed that patients with multiple diseases have high risks of ED visits, where such information typically is recorded in unstructured medical notes during the diagnosis. To improve prediction performance, this study extracts diseases from unstructured subjective, objective, assessment, and plan notes, along with the *ICD-10* from EHR, and then calculates the CCI score [14] to indicate the patient’s condition.

### Feature Embedding

Inputs to ML models must be numerical, and therefore, nonnumeric features usually need to be encoded or embedded before feeding them into the model. Feature embedding or feature encoding serves as a bridge between raw data and model inputs, enabling algorithms to operate efficiently on transformed data. Since feature embedding is as critical as feature selection for building an efficient model, this study designs 2 feature encoding schemes to evaluate the impact of feature embedding and to identify the most suitable one for the prediction model. Table 5 explains the studied feature encoding schemes in detail.

**Table 5.** Studied encoding methods.

Feature	Range	Description
Encoding E_A_		
Age^a^ (years)	0-5	0: <41; 1: 41-50; 2: 51-60; 3: 61-70; 4: 71-80; 5: >80
MAP^b^ (mmHg)	0-2	0: <70; 1: 70-100; 2: >100
HBP^c^ (mmHg)	0-2	0: <90; 1: 90-120; 2: >120
LBP^d^ (mmHg)	0-2	0: <60; 1: 60-80; 2: >80
Pulse rate (bpm)	0-2	0: <60; 1: 60-100; 2: >100
Encoding E_B_		
Age^e^ (years)	0-2	0: <41; 1: 41-65; 2: >65
MAP (mmHg)	0-1	0: 70-100; 1: otherwise
HBP (mmHg)	0-1	0: 90-120; 1: otherwise
LBP (mmHg)	0-1	0: 60-80; 1: otherwise
Pulse Rate (bpm)	0-1	0: 60-100; 1: otherwise
Temperature (°C)	0-1	0: 35-38; 1: otherwise
SPO_2_ (%)^f^	0-1	0: 95-100; 1: otherwise
Lab test count	0-∞	Count for the total number of the ordered lab tests
Medicine count	0-∞	Count the total number of the ordered medicines
ICD-10^g^	0-22	According to [15]

a Based on Charlson Comorbidity Index.

b MAP: mean arterial pressure.

c HBP: high blood pressure.

d LBP: low blood pressure.

e Based on Chen et al [16].

f SpO_2_: saturation of peripheral oxygen.

g *ICD-10*: *International Statistical Classification of Diseases, Tenth Revision*.

The studied data contains a mix of numerical and categorical data. Even though the studied structured data is mostly numerical, its values represent different meanings. Vital signs are in numerical form but can be interpreted as categorical variables, as vital signs usually have a normal range. For example, HBP can be tiered into 3 levels—0 (low HBP), 1 (normal HBP), and 2 (high HBP)—as shown in the first encoding method of Table 5. On the other hand, for the ML model, low HBP and high HBP both imply the patient’s HBP is not in the normal range, and there might not be much difference between too high and too low. Therefore, the HBP feature value can be interpreted as normal or nonnormal. As expressed in the second encoding method of Table 5, it is categorized into 2 types: 0 for normal HBP and 1 for HBP not in the normal range. Those numerical features not listed in the table are applied in their original numerical values to the ML models.

**Figure 3.**
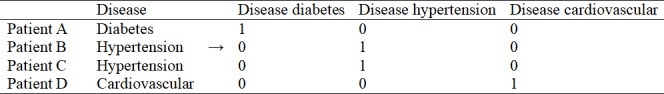
Pandas’ one-hot embedding method.

When working with categorical data, such as disease names, it is critical to convert them into a numerical format so that ML algorithms can understand them. One-hot encoding is commonly used for encoding categorical variables. This study adopts an improved one-hot encoding scheme, which presents a group of related categories together, as illustrated in Figure 3.

### Class Imbalance

Training an effective model requires a balanced or near-balanced class distribution. However, the datasets collected from real-world environments are typically imbalanced, as most ED visits are non-URV cases, which constitute the major class. Sampling is a process of resampling data to improve dataset distribution, whereas oversampling is a process of increasing the sampling rate of the minority class, while undersampling reduces the sampling rate of the majority class.

Oversampling techniques applied to the minority class, which are widely used to address data imbalance issues, may introduce several significant challenges, particularly in the medical domain. For instance, methods such as random duplication of existing data can lead to model overfitting, as the model may overly rely on a restricted subset of the input. Furthermore, approaches like SMOTE or other algorithms that dynamically generate synthetic data may pose ethical and legal concerns, as the generated data could be false or misleading, ultimately negatively impacting the prediction model. In contrast, random undersampling can effectively reduce a considerable number of non-URV records, resulting in a balanced dataset with minimal information loss.

Based on the aforementioned analysis, this study adopts random undersampling to achieve a balanced dataset while minimizing the risks associated with oversampling techniques. Given that oversampling may introduce model overfitting and ethical concerns due to synthetic data generation, random undersampling presents a more reliable approach by reducing the number of non-URV records without compromising data integrity. This method ensures that the dataset maintains a proportional distribution between classes, thereby enhancing the model’s generalizability and mitigating bias.

### Prediction Model

To identify the optimal ML algorithms tailored for the proposed framework, this study evaluates various classification algorithms commonly used in related literature. These include DT, LR, random forest (RF), support vector machine (SVM), one-class support vector machine (OCSVM), grid search cross-validation (GSCV), multilayer perceptron (MLP), convolutional neural network (CNN), tree-based pipeline optimization tool, and long short-term memory (LSTM).

Hyperparameter fine-tuning is a critical process that directly influences model performance. To determine the optimal hyperparameters for each algorithm, multiple combinations of hyperparameter sets are independently evaluated.

Additionally, the different FSs and feature encoding methods described earlier are assessed to construct an effective URV prediction model.

To develop and evaluate the prediction models, the dataset was randomly partitioned into training and testing sets with a ratio of 8:2. This process was repeated 10 times with different random splits, and the final performance metrics were reported as the average across these 10 runs to ensure robustness and reduce variance due to data partitioning.

### System Interface

**Figure 4.**
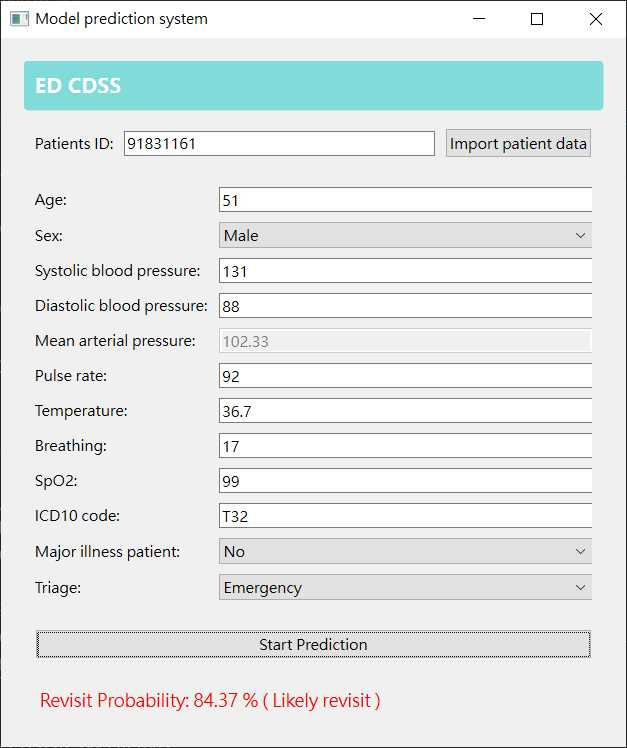
Graphical user interface of the proposed system as a standalone application.

The proposed system is designed to provide a straightforward and user-friendly interface, enabling physicians to efficiently enter medical records, as illustrated in Figure 4.

The interface consists of 2 primary sections: the clinical data input section and the model output section. The clinical data input section includes structured data fields for entering numerical values, as well as dropdown menus for selecting categorical features. The use of dropdown menus minimizes potential input errors and reduces input time, thereby enhancing data entry efficiency. Once the required data are entered, they are fed into the pretrained prediction model upon submission. The prediction model then analyzes the clinical input data to determine whether a patient is at risk of revisiting the ED within 72 hours. The prediction output provides the likelihood of a revisit along with the model’s decision, offering physicians a clear assessment of patient risk.

The intuitive design of the interface increases physician adoption and usability, facilitating seamless integration into clinical workflows. By streamlining data entry and automating risk assessment, the system improves decision-making efficiency while minimizing potential input errors. The straightforward structure ensures that physicians can quickly interpret and act upon prediction results, ultimately supporting more effective ED management.

## Results

### Overview

According to past studies and our preliminary study, several factors might impact training efficiency. Therefore, this study conducts 2 experiments in order to obtain an efficient URV prediction model for ED by evaluating different ML models, FSs, and encoding schemes. The first experiment investigates the prediction performance of traditional and modern ML algorithms by applying 2 types of FSs (a complete set vs a subset of selected features), and the second experiment evaluates the impact of feature encoding.

**Figure 5.**
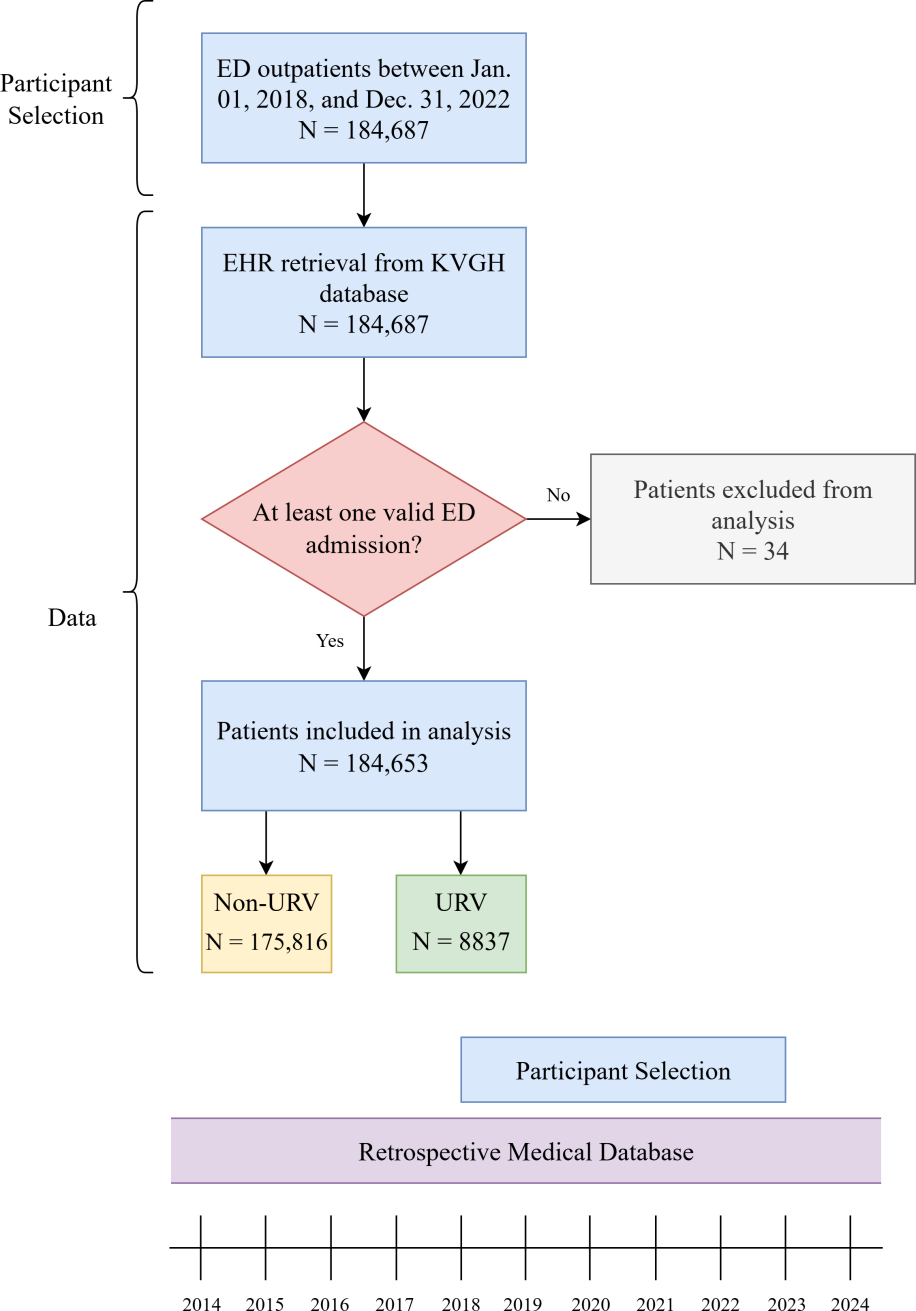
Participant selection, data, and timeline. ED: emergency department; EHR: electronic health record; KVGH: Kaohsiung Veterans General Hospital; URV: unscheduled return visit.

Figure 5 illustrates the process of data selection, data filtering, and the study timeline for a retrospective medical study. The selection process begins with 184,687 ED patients treated between January 2018 and December 2022. EHRs were retrieved from the KVGH database for these patients. To ensure data validity, a screening criterion was applied, requiring at least one valid ED admission. As a result, 34 patients were excluded, leaving a final study cohort of 184,653 patients for analysis.

All experiments were performed on a personal computing workstation configured with a 12th Generation Intel Core i7 CPU, 32 GB of RAM, an NVIDIA RTX 3060 dedicated GPU, and a 512 GB solid-state drive.

### Performance Measurement

Model performance is evaluated using metrics such as accuracy, recall, and the area under the receiver operating characteristic curve (AUROC). AUROC is a widely used metric for assessing the effectiveness of binary classification models, as the receiver operating characteristic curve illustrates the trade-off between the true positive rate and the false positive rate across various decision thresholds. AUROC values range from 0.5 to 1.0, where a value of 0.5 indicates that the model performs no better than random chance in distinguishing between the 2 classes.

Certain models may achieve a high recall rate but low accuracy, while others may exhibit high accuracy but low recall. Neither type of model is suitable for reliable prediction. Since AUROC accounts for both aspects of classification performance, this study selects AUROC as the primary metric for evaluating prediction performance.

### Performance Evaluation of ML Models for URV Prediction

This section investigates the predictive performance of various ML algorithms using different FSs: FS_A_, FS_B_, and FS_C_. These ML algorithms iteratively learn important patterns from the input data and adjust feature weights accordingly. However, an excessive number of features can introduce noise and negatively impact model training, highlighting the necessity of a feature selection process during training. A carefully selected subset of key features may yield more effective performance. Therefore, in addition to evaluating various ML models, this experiment also examines the effectiveness of the selected features.

Table 6 summarizes the results, comparing 2 categories of ML models: traditional ML and modern ML. In terms of predictive modeling, modern ML algorithms generally require longer training times but produce better prediction models compared with traditional ML algorithms, with the exception of LSTM. The best-performing modern ML model (CNN) surpasses the best-performing traditional ML model (LR) and outperforms all other models, achieving superior performance with a reasonable training time.

**Table 6.** The results of experiment 1.

Model, encoding, and FS^a^	Accuracy	Recall	AUROC^b^	Training time (sec)
Traditional ML models				
DT^c^				
E_A_				
FS_A_	0.290	0.908	0.588	0.02
FS_B_	0.857	0.283	0.581	0.03
FS_C_	0.805	0.362	0.592	0.02
LR^d^				
E_A_				
FS_A_	0.624	0.602	0.636	0.07
FS_B_	0.547	0.614	0.609	0.07
FS_C_	0.628	0.602	0.615	0.07
OCSVM^e^				
E_A_				
FS_A_	0.348	0.871	0.600	1.19
FS_B_	0.140	0.893	0.502	0.48
FS_C_	0.169	0.894	0.518	0.40
RF^f^				
E_A_				
FS_A_	0.628	0.636	0.632	1.11
FS_B_	0.629	0.548	0.590	0.53
FS_C_	0.633	0.619	0.626	0.94
SVM^g^				
E_A_				
FS_A_	0.432	0.689	0.556	4.99
FS_B_	0.411	0.725	0.562	4.06
FS_C_	0.678	0.558	0.620	3.76
Modern ML^h^ models				
CNN^i^				
E_A_				
FS_A_	0.565	0.718	0.705	48.61
FS_B_	0.629	0.562	0.647	48.36
FS_C_	0.646	0.620	0.698	42.74
GSCV^j^				
E_A_				
FS_A_	0.630	0.640	0.639	864.23
FS_B_	0.645	0.560	0.600	888.46
FS_C_	0.693	0.577	0.637	882.76
LSTM^k^				
E_A_				
FS_A_	0.043	0.989	0.568	52.35
FS_B_	0.503	0.538	0.526	50.95
FS_C_	0.608	0.565	0.633	51.27
MLP^l^				
E_A_				
FS_A_	0.568	0.719	0.692	13.06
FS_B_	0.803	0.321	0.626	8.32
FS_C_	0.649	0.613	0.691	14.87
TPOT^m^				
E_A_				
FS_A_	0.642	0.685	0.663	183.23
FS_B_	0.653	0.556	0.607	148.94
FS_C_	0.684	0.614	0.650	177.05

a FS: feature set.

b AUROC: area under the receiver operating characteristic.

c DT: decision tree.

d LR: logistic regression.

e OCSVM: one-class support vector machine.

f RF: random forest.

g SVM: support vector machine.

h ML: machine learning.

i CNN: convolutional neural network.

j GSCV: grid search cross-validation.

k LSTM: long short-term memory.

l MLP: multilayer perceptron.

m TPOT: tree-based pipeline optimization tool.

Among these combinations, LR with FS_A_ outperforms other traditional ML algorithms in terms of AUROC, which is indicative of overall model performance. The evaluated traditional ML models (DT, SVM, and OCSVM) exhibit diverse prediction performances, with some demonstrating high accuracy but low recall, while others show high recall but low accuracy. Notably, LR and RF deliver stable performance across both metrics.

In contrast, most modern ML models yield stable prediction performance, except for LSTM. While LSTM is highly suitable for time-series prediction, it is not appropriate for the current dataset, leading to the poorest performance among all modern ML and traditional ML models, except OCSVM with FS_B_. Given that the studied dataset comprises only 24 distinct features, simpler algorithms such as DT and LR in traditional ML and CNN in modern ML outperform more complex models.

The superior performance of simpler models on this dataset can be attributed to the nature of the data. Complex algorithms, such as LSTM, typically require larger datasets with extensive FSs to effectively capture patterns and avoid overfitting. When applied to datasets with fewer features, these models are more prone to overfitting, leading to reduced generalization and poorer performance. In contrast, simpler algorithms, which rely on fewer parameters and are less prone to overfitting, can effectively handle smaller datasets, making them more suitable in such cases.

### Impact of Feature Set on Model Performance

With respect to feature selection, the complete FS (FS_A_) generally builds better prediction models compared with the subsets (FS_B_ and FS_C_) according to the results in Table 6. The discrepancies in AUROC between FS_A_ and FS_C_ fall within ±1, suggesting that the subset contains critical determinants for URV prediction. Both FS_B_ and FS_C_ primarily consist of vital signs and age, indicating that these are key features for URV prediction. Moreover, FS_C_ incorporates medical notes and outperforms FS_B_ across all evaluation models, highlighting the importance of unstructured data in enhancing predictive performance.

### Comparison of Training Time Across ML Models

Regarding training time, traditional ML algorithms generally require less time to complete their training phases compared with modern ML algorithms. GSCV, for instance, requires at least 860 seconds to build a prediction model but does not achieve the best detection results. In contrast, CNN, which yields the best performance, completes its training phase in an average of 48 seconds, while MLP, the second-best algorithm, requires less than 15 seconds on average. As MLP is a simplified version of CNN, it is expected that CNN takes longer training time than MLP. Notably, both MLP and CNN outperform other models, with only minor discrepancies observed between their performances.

### Performance Evaluation of ML Models With Different Feature Encoding

This section investigates the impact of feature encoding on model training. This experiment evaluates 2 encoding methods (E_A_ and E_B_) on the 2 best prediction models (MLP and CNN) with the best FS (FS_A_) obtained from the previous experiment and another feature subset (FS_C_), since FS_C_ outperforms FS_B_ across all evaluation models. The studied encoding methods are described in the previous section and outlined in Table 5. FS_C_ includes FS_B_ and some other relevant features, as shown in Table 4, in order to investigate the encoding impact on different types of features.

Table 7 lists the evaluation results, where the boldface figure indicates the best of a given performance measurement. The results demonstrate that the encoding method has a certain impact on model training. A simplified encoding E_B_ performs better than E_A_. That is, categorizing vital signs into 2 types (normal and abnormal) can better represent the meaning of the features than the 3 types (low, normal, and high); converting *ICD-10* into 22 categories defined by World Health Organization can better represent the meaning of the diseases; age classified into 3 categories (0: <41 years; 1: 41-65 years; 2: >65 years) is better than the 6 categories defined by CCI (0: <41 years; 1: 41-50 years; 2: 51-60 years; 3: 61-70 years; 4: 71-80 years; 5: >80 years).

**Table 7.** The results of experiment 2.

Model and FS^a^	Encoding	Accuracy	Recall	AUROC^b^
MLP^c^
FS_A_	E_A_	0.568	0.719	0.692
FS_A_	E_B_	0.602	0.724	0.734
FS_C_	E_A_	0.649^d^	0.613	0.694
FS_C_	E_B_	0.620	0.652	0.698
CNN^e^
* *FS_A_	E_A_	0.565	0.718	0.705
FS_A_	E_B_	0.606	0.740^d^	0.747^d^
FS_C_	E_A_	0.646	0.620	0.698
FS_C_	E_B_	0.625	0.673	0.715

a FS: feature set.

b AUROC: area under the receiver operating characteristic curve.

c MLP: multilayer perceptron.

d The best of the given performance measurement.

e CNN: convolutional neural network.

### Model Parameters

The CNN model architecture consists of a repeated block structure, composed of two 1D convolutional layers followed by a 1D max-pooling layer. This block is repeated 5 times, after which the output is passed to a fully connected layer and finally mapped to the probability of a URV.

Each convolutional layer uses 64 filters with a kernel size of 3 and uses the rectified linear unit as the activation function. The max-pooling layer is configured with a pool size of 2.

For model training, the number of epochs was set to 100, and an early stopping mechanism was implemented to prevent overfitting, with a patience value of 25. The model was optimized using the Nadam optimizer with a batch size of 256. A dynamic learning rate adjustment strategy was applied, where the learning rate was reduced by a factor of 0.5 if no improvement was observed for 5 consecutive epochs. The minimum learning rate was set to 1^e–4^, with epsilon set to 0.0001.

## Discussion

### Principal Findings

This study proposes an ML-assisted system for predicting patients at risk of revisiting the ED, leveraging features extracted from both structured and unstructured medical data. The structured data consists of patients’ EHRs, while the unstructured data includes medical notes. The findings indicate that the conventional trade-off between training time and prediction performance may not be universally applicable. While traditional ML models require significantly less training time, modern ML models demand at least twice as much, if not more. When computational resources or training time are constrained, traditional ML models such as LR can serve as effective prediction tools; however, when such limitations are not a major concern, CNN is recommended. Additionally, while incorporating a broad range of relevant features enhances predictive performance, a subset of key features may have a dominant impact on model outcomes. In particular, vital signs and age are identified as critical predictors for URV, and properly encoding features into the appropriate categories can further improve model efficiency.

In this study, 2 encoding strategies (E_A_ and E_B_) were evaluated. The experimental results indicate that models trained on datasets encoded using the E_B_ approach consistently outperform those trained with the E_A_ approach. This performance difference can be attributed to the characteristics of the respective encoding methods. Although E_B_ uses a relatively simpler encoding scheme, it distinctly classifies variables into 2 well-defined categories: normal and abnormal. In contrast, E_A_ uses a 3-category system— “too low,” “normal,” and “too high.” However, the categories “too low” and “too high” both represent abnormal conditions and may not be sufficiently distinguishable in practice. This lack of distinction could introduce ambiguity for the predictive model, thereby adversely affecting its performance.

For practical implementation, AUROC is used to represent the overall performance of a model. Models with higher AUROC values are associated with fewer false alerts while maintaining an acceptable true positive rate. According to the experimental results, among traditional ML models, LR demonstrates the best performance in predicting URV, achieving an AUROC of 0.636. Among modern ML models, CNN outperforms its counterparts in terms of AUROC values.

In scenarios where capturing all URV cases is prioritized, recall serves as a critical performance indicator. Among traditional ML models, DT achieves the highest recall rate of 0.908, while among modern ML models, LSTM achieves the highest recall value of 0.989. However, it is noteworthy that both DT and LSTM exhibit low accuracy values, indicating that these models tend to generate a considerable number of false alerts.

### Limitations

A notable finding is that, although advanced CNN-based models such as LSTM typically outperform CNN in most ML applications, this advantage does not necessarily hold in URV prediction for ED visits. One possible explanation is that ED visits often lack prior knowledge of a patient’s visit history, which limits the predictive capacity of sequential models like LSTM. Furthermore, the absence of sufficient medical history may constrain the overall performance of ML-based prediction models. Future research could explore the integration of patients’ comprehensive medical histories to enhance predictive accuracy. Additionally, the diverse causes of ED revisits present a challenge for ML models, as they make it difficult to distinguish patterns among different revisit cases. Investigating ML-based predictions for specific revisit causes may further refine model performance.

Given the nonstandardized nature of medical notes, this study transforms the unstructured textual content into corresponding *ICD-10* codes. This structured representation has been shown to enhance the predictive performance of the model. However, medical notes possess a wide array of clinically relevant information beyond *ICD-10* codes, including chief complaints, prior treatments, and allergy histories. Therefore, the application of advanced natural language processing techniques may further improve predictive outcomes by enabling the extraction of richer features from unstructured clinical text.

### Comparison With Prior Work

Previous studies mostly focused on general revisits, while studies specifically addressing ED revisits were relatively understudied. Most existing work (Vest and Ben-Assuli [11]) considered a long prediction window, such as 30 days, while revisit time frames are likely shorter, for example, 72 hours [12]. Most existing prediction models selected features from merely one type of EHR data, rarely considering both types of patients’ medical data: structured EHR and unstructured medical notes. Prior studies [6-8] that applied ML prediction models primarily evaluated 1 or 2 models and rarely analyzed the performance of multiple prediction models or various FSs.

A similar study by Guo et al [8] on ED revisits incorporated both structured and unstructured medical records by converting them into semantic patterns and developing a customized bidirectional encoder representations from transformers (BERT) model, referred to as BlueBERT, to predict URVs. The issue of data imbalance, with URV cases accounting for only 2.22% of their studied dataset, was addressed using random undersampling techniques. Their experimental results demonstrated the superiority of BlueBERT compared with other models, such as KNN, RF, and XGB, in terms of AUROC.

To the best of our knowledge, our study is one of the few attempts to combine 2 types of patients’ medical data (structured and unstructured) to identify unplanned revisits to the ED and is the first study that conducts a comprehensive performance evaluation of traditional as well as modern ML classification models, where the traditional ones include LR, RF, SVM, and OCSVM and the modern ones include GSCV, MLP, CNN, tree-based pipeline optimization tool, and an improved CNN LSTM. Furthermore, this study analyzes the importance of feature selection and the impact of feature embedding on the efficiency of model training.

### Conclusions

This study evaluates both traditional ML and modern ML models for predicting the URVs in ED and examines the impacts of feature selection and feature encoding on model training. The evaluation concludes the following findings: (1) adopting an appropriate model is important for a targeted problem, (2) not all the MLL models are superior to traditional ML ones, (3) an advanced modern ML model might not yield better performance than a basic modern ML model (such as CNN) or a traditional ML (such as LR), (4) a complicated algorithm requiring long training time (such as GSCV) might not construct an efficient prediction model, (5) feature selection is relevant to build an efficient model, (6) finding key features is critical for interpreting the prediction results, (7) feature encoding affects the efficiency of model training, and (8) an encoding scheme which better represents the meaning of the features could yield a better prediction model.

## Abbreviations

AUROC: area under the receiver operating characteristic
BERT: bidirectional encoder representations from transformers
CCI: Charlson Comorbidity Index
CNN: convolutional neural network
DT: decision tree
ED: emergency department
EHR: electronic health record
FS: feature set
GSCV: grid search cross-validation
HBP: high blood pressure
*ICD-10*: *International Statistical Classification of Diseases, Tenth Revision*
IRB: institutional review board
KVGH: Kaohsiung Veterans General Hospital
LR: logistic regression
LSTM: long short-term memory
ML: machine learning
MLP: multilayer perceptron
OCSVM: one-class support vector machine
RF: random forest
SVM: support vector machine
URV: unscheduled return visit
